# Comparison of the Relationship Between Sexual Function, Marital Adjustment, and Life Satisfaction in Diabetic and Non-Diabetic Women

**DOI:** 10.30476/ijcbnm.2020.84993.1244

**Published:** 2020-10

**Authors:** Roghayeh Mehdipour-Rabori, Mahdieh Alinejad Dehsheakhi, Esmat Nouhi, Monirsadat Nematollahi

**Affiliations:** 1 Nursing Research Center, Kerman University of Medical Science, Kerman, Iran; 2 Department of Nursing and Medical Education, School of Nursing and Midwifery, Kerman University of Medical Sciences, Kerman, Iran; 3 Department of Pediatrics and Neonatal Intensive Care Nursing, School of Nursing and Midwifery, Kerman University of Medical Sciences, Kerman, Iran

**Keywords:** Sexual function, Marital relationship, Diabetic women, Personal Satisfaction

## Abstract

**Background:**

Sexual function is important for diabetic women because it has a special effect on the quality of life. This study was conducted to compare the relationship of sexual function, marital adjustment, and life satisfaction between diabetic and non-diabetic women.

**Methods:**

This cross-sectional study was done in Kerman, Iran, from August 2018 to November 2019. The study sample included 300 diabetic women and 300 non-diabetic women. Data gathering tools included a demographic questionnaire, Rosen female sexual function index (FSFI), Spanier Dyadic Adjustment Scale (DAS), and Diner satisfaction with life scale. Data were analyzed through SPSS 15, using descriptive statistics, independent-t test, ANOVA, and Pearson tests. The significance level was considered 0.05.

**Results:**

The mean scores of marital adjustment, female sexual function index, and satisfaction with life scales in diabetic women were 90.98±23.33, 19.04±9.77, and 13.4±3.21; also, they were 120.34±33.34, 27.82±10.17, and 16.3±5.89 in non-diabetic women, respectively. Statistically significant differences were found between the scores of marital adjustment (P=0.001), female sexual function index (P=0.001), and satisfaction with life (P=0.001) in diabetic and non-diabetic women. The female sexual function index was correlated with life satisfaction and marital adjustment.

**Conclusion:**

Diabetic women experience sexual dysfunction because of their special condition, which negatively influences life satisfaction and marital adjustment. Healthcare providers should pay much more attention to this issue. They can provide educational packages on sexual issues for diabetic women. They should also support these women and their spouses to improve their quality of life.

## INTRODUCTION

Diabetes causes major issues in the patients’ general health, ^1^ affecting nearly all aspects of human life and changing the lifestyle of diabetic patients. On the other hand, diabetes prevalence is rapidly increasing worldwide; it is estimated that 550 million people will have suffered from diabetes by 2030. ^2^ According to available statistics, the prevalence of diabetes mellitus in people aged 25–64 years was 7.7% (2 million people) in Iran in 2005, and it will have been around 5.2 million by 2025, ^3^ and 14 million by 2030. ^4^

In the long term, diabetes leads to minor complications such as retinopathy, neuropathy, and major vessel complications, myocardial infarction, angina pectoris, and stroke. ^5^ Neurovascular disorders caused by diabetes can lead to structural and functional changes in the female reproductive system and disturb sexual response. Hyperglycemia decreases vaginal mucus hydration resulting in decreased lubrication and elevated pain. Moreover, hyperglycemia is associated with a higher risk of reproductive system infection, which will disturb the vaginal mucus and thus leads to dyspareunia. ^6^ Sexual desire is a complex process coordinated by neural, vascular, and endocrine systems. Sexual function is affected by familial, social, religious beliefs, health status, and personal experience. Many women, especially diabetic women, are known to experience sexual dysfunction. ^7^ The worldwide prevalence of sexual dysfunction is 30-78 percent. Women’s sexual dysfunction is a disorder in sexual desire, orgasm, arousal, and sexual pain, which can lead to significant personal and interpersonal tension. ^8^ Alteration in blood sugar level can lead to fatigue and irritability in some diabetic women and a decreased desire for sexual intercourse. Vaginal dryness can lead to dyspareunia. ^9^ A study was done in Iran to compare the sexual function of diabetic and non-diabetic women in 2009. It was concluded that the sexual function of diabetic women was significantly lower than that of the non-diabetic women in terms of sexual desire, arousal, vaginal lubrication, and orgasm. ^10^ However, few studies have linked the sexual function to marital adjustment, but researches have shown that marital adjustment has a significant impact on the quality of life. ^11^ Marital adjustment is the accommodation of spouses to each other, and intimacy and cooperation are achieved through common understanding. ^12^ Marital adjustment is the result of general satisfaction with marital life, satisfaction with the sexual relationship, and emotional satisfaction. ^13^ One study showed a significant relationship between marital satisfaction, marital adjustment, and diabetes mellitus II. ^14^ Marital adjustment and life satisfaction are probably related. Life satisfaction is a subjective and cognitive evaluation of the personal life as a whole. Life satisfaction and happiness are mental satisfaction indexes and aspects of mental health. ^15^ Increased life satisfaction is an important factor in the life of patients with diabetes, indicating proper therapeutic effects and improvement of the health status of the patients. ^16^

Research showed life dissatisfaction, low self-esteem, and poor health in diabetic patients. ^17^ A study done on 793 diabetic patients in Jordan showed no significant difference between diabetic men and women in terms of life satisfaction, self-efficacy, and disease management. Another study reported higher life satisfaction in patients with higher self-efficacy and better understanding. ^18^

Although various studies have addressed sexual function, life satisfaction, and marital adjustment in different patients, the researchers found no study on the three components simultaneously. Iranian women may be ashamed of talking about sexual dysfunction. Since women play a crucial role in families, their physical, mental, and sexual health affects the family health. Suppression of the natural needs of women will have detrimental effects on family relationships and happiness. Owing to the fact that a healthy sex is a natural right of every woman, and most women with diabetes are young and middle-aged, health care providers should recognize the difference in the sexual function between diabetic and non-diabetic women. Thus, this study was conducted to compare the relationship of sexual function, marital adjustment, and life satisfaction between diabetic women and non-diabetic women.

## MATERIALS AND METHODS

This cross-sectional study was conducted in Kerman, Iran, from August 2018 to November 2019. The sample size included 300 non-diabetic women and 300 diabetic women referring to diabetes clinics in Kerman, Iran. A pilot study was performed to determine the sample size with α=0.05 and study power of 80%. 

The inclusion criteria of this study were women aged 20-45 years with no history of cardiac or reproductive diseases, endometriosis, uterine fibroids, gynecologic cancers, human immunodeficiency virus (HIV)/acquired immunodeficiency syndrome (AIDS), interstitial cystitis, polycystic ovary syndrome (PCOS), and sexually transmitted diseases (STDs) that would affect their sexual function according to their medical records. In addition, diabetic women had to be under treatment for one year and had to take at least one medication for their diabetes control. Moreover, being able to speak Persian and not suffering from psychological disorders were other inclusion criteria of this study. The exclusion criterion was incomplete questionnaires.

The researchers matched two groups in terms of age, contraceptive methods, and income. The women were informed of the study objectives, and the written informed consent was obtained before the participants filled in the questionnaires. In the case of illiterate participants, the questionnaires were filled in orally. Four questionnaires were used for data collection: demographic information questionnaire, Rosen female sexual function index (FSFI), Spanier Dyadic Adjustment Scale (DAS), and satisfaction with life scale (SWLS). Demographic information questionnaire included age, job, income level, education level, course of diabetes, number of children, contraception method, and diabetes medications.

FSFI questionnaire was designed in 2000, ^19^ and Mohammadi et al. (2008) translated it into Persian for psychometric measurement. ^20^ This questionnaire includes 19 items assessing the women’s sexual function in 6 independent domains of desire, arousal, lubrication, orgasm, satisfaction, and pain. Each item is scored from 0 to 5. The score of each domain is calculated by adding the scores of each item multiplied by its factor. The total score is calculated by summing up the scores of these six domains. Thus, higher scores show better sexual function. The maximum score for each domain is six, and the maximum total score is 36. The reliability of this questionnaire for each section has been thoroughly assessed in different studies, and Cronbach’s Alphas of 0.70 and 0.89 have been reported. ^20^ Fakhri in Iran assessed the reliability and validity of this questionnaire in 2011, so that the reliability was between 0.73 and 0.86, and the validity was between 0.72 and 0.90. ^21^

DAS is a self-report measure designed by Graham B. Spanier in 1976. ^22^ Isanezhad prepared the Persian version of DAS in 2012. ^23^ This questionnaire includes 32 items with four dimensions: Dyadic Consensus, Dyadic Satisfaction, Dyadic Cohesion, and Affectional Expression. Items 1-15 were scored based on a six-point scale from ‘Always Agree’ to ‘Always Disagree’ with each item scored from 0 to 5. Items 16–22 were scored based on a different 6-point scale from ‘All the Time’ to ‘Never’ with each item scored from 0 to 5. Item 23 was scored based on a 5-point Likert scale from ‘Every Day’ to ‘Never,’ with each item scored from 0 to 4. Item 24 was scored based on a 5-point Likert scale from ‘All of Them’ to ‘None of Them’ with each item scored from 0 to 4. Items 25–28 were scored based on a 6-point scale from ‘Never’ to ‘More Often’ with each item scored from 0 to 5. Items 29–30 were scored based on a dichotomous scale of ‘Yes’ or ‘No’. Item 31 was scored based on a 7-point Likert scale from ‘Extremely Unhappy’ to ‘Perfect,’ and the scoring was 0-6, and item 32 included 6 options and asked the participants to choose the most relevant response (0-5).

The subscales were scored by summing the items of each subscale. The total score was obtained by summing all the subscale scores. Scores of this scale ranged from 0 to 151 and scores above 100 indicated high marital adjustment. The internal consistency coefficient of this scale was 96%. ^22^ Reliability and validity of the Iranian version of the questionnaire were confirmed, and the Cronbach’s alpha coefficient was 0.79. ^23^

SWLS was used to assess life satisfaction. This scale was developed in 1985 and included five questions. ^24^ The answers were based on the Likert Scale (1: strongly disagree, 2: disagree, 3: no idea, 4: agree, 5: strongly agree). The scores of 5-10 showed poor life satisfaction, scores of 10-15 indicated moderate life satisfaction, and scores above 15 indicated high life satisfaction. Diner et al. (1985) reported the reliability and validity of this scale for life satisfaction with Cronbach’s Alpha=0.80. ^24^ Bayani translated the scale in 2007 and assessed the validity and reliability. The reliability was 0.83. ^25^

This study was confirmed by the ethics committee of Kerman University of Medical Sciences and obtained the code of ethics IR.KMU.REC.1396.2376. The objectives of the study were explained to the participants, and written informed consent was taken. 

When the participants filled in the forms, the data were analyzed using SPSS 15 software. P value&lt;0.05 was considered significant in all the tests. Descriptive statistics (frequency, percent, mean and standard deviation) were used to describe the demographic characteristics. independent-t test and ANOVA tests were used to determine the difference between the two groups. Pearson was used to determine the correlation between sexual satisfaction, marital adjustment, and life satisfaction.

## RESULTS

The results of this study showed that most participants were 36-45 years old (53.8%). Table 1 shows the demographic information of the participants. The dropout probability was 0.4%, but the samples were easy to access, and the researchers replaced new ones. 

**Table 1:** Demographic information of the diabetic and non-diabetic women

Variable		Diabetic group	Non-diabetic group	P value
		N (%)	N (%)	
Age	20-35	140 (46.70)	137 (45.63)	0.48*
	36-45	160 (53.30)	163 (54.57)	
Job	Housewife	229 (76.41)	210 (70.00)	0.96**
	Employee	48 (16.00)	52 (17.37)	
	Self-employed	23 (7.59)	38 (12.63)	
Level of education	Illiterate	100 (33.29)	97 (32.38)	0.10 **
	Diploma	149 (49.71)	152 (50.62)	
	Bachelor	46 (15.41)	48 (16)	
	Above Bachelor	5 (1.59)	3 (1)	
Contraception	Condom	49 (16.44)	46 (15.35)	0.21**
	Oral contraceptive	18 (6.00)	21 (7.00)	
	Natural	144 (48.00)	138 (46.00)	
	Tubal ligation	52 (17.31)	54 (18.00)	
	Other methods	37 (12.25)	41 (13.65)	

* Independent-t test;

** ANOVA

The mean total score of the sexual function of diabetic women was 19.04±9.77, and it was 27.82±10.17 in non-diabetic women. A significant difference was found in the mean total score of sexual function between diabetic and non- diabetic women (P&lt;0.001) (Table 2).

**Table 2:** Comparison of mean sexual function between diabetic and non-diabetic women

Variable	Diabetic women Mean±SD	Non-diabetic women Mean±SD	P value*
Desire	3.00±1.05	4.01±1.06	0.01
Arousal	2.66±1.46	5.00±2.01	0.001
Lubrication	2.83±1.55	4.18±1.92	0.002
Orgasm	3.18±1.81	5.11±2.13	0.02
Satisfaction	3.21±1.64	5.00±1.02	0.001
Pain	4.16±2.26	4.52±2.03	0.05
Total score	19.04±9.77	27.82±10.17	0.001

* Independent-t test

The mean total score of life satisfaction in diabetic women was 13.4±3.21, and it was 16.3±5.89 in non-diabetic women. A significant difference was observed in the mean total score of life satisfaction between diabetic and non- diabetic women (P&lt;0.001).

The total scores of marital adjustment in diabetic women ad non-diabetic women were 90.98±23.33, 120.34±33.34, respectively. The independent t-test showed a significant difference in the marital adjustment between the two groups. Table 3 shows the highest and lowest scores in the two groups (Table 3).

**Table 3:** Comparison of mean marital adjustment between diabetic and non-diabetic women

Variable	Diabetic women Mean±SD	Non-diabetic women Mean±SD	P value*
Dyadic Satisfaction	31.11±7.63	42.98±8.43	0.001
Dyadic Cohesion	16.62±2.47	23.74±7.62	0.001
Dyadic Consensus	36.34±11.64	42.28±14.54	0.02
Affectional expression	6.91±1.59	11.34±2.75	0.03
Total Marital Adjustment	90.98±23.33	120.34±33.34	0.001

* Independent-t test

Table 4 shows the relationship between sexual function, life satisfaction, and marital adjustment. 

**Table 4:** Correlation among marital adjustment, sexual function, and life satisfaction

Variable	Diabetic women		Non-diabetic women	
	r	P value*	r	P value*
Marital adjustment	0.38	0.001	0.28	0.01
Sexual function				
Sexual function	0.29	0.01	0.25	0.01
Life satisfaction				
Marital adjustment	0.49	0.001	0.53	0.001
Life satisfaction				

* Pearson

## DISCUSSION

The present study compared the sexual function, marital adjustment, and life satisfaction in diabetic women and non-diabetic women in 2018-2019. The results of the study indicated that the sexual function, life satisfaction, and marital adjustment in diabetic women were lower than those in the non-diabetic women. In addition, the results showed a significant positive relationship between the sexual function, marital adjustment, and life satisfaction in diabetic and non-diabetic women.

In the present study, the mean total score of sexual function in diabetic women was lower than that of the non-diabetic women. The studies indicated poor sexual function of women with diabetes compared with the non-diabetic women. ^26 , 27^ One study in Iran reported that more than half of the diabetic women suffered from sexual dysfunction. ^28^ Sexual dysfunction was not only seen in women with type 2 diabetes, but also women with type 1 diabetes suffered from sexual dysfunctions. ^26^ Furthermore, a study showed that the sexual function in women of reproductive age with type 2 diabetes was significantly lower than that of the non-diabetic women. ^29^

The results of another study on diabetic women of reproductive age and healthy women showed that the most common symptom in diabetic patients was lack of libido followed by vaginal dryness, and orgasmic dysfunction, and there was a significant difference in all areas of sexual function between the two groups. ^30^ The results of the studies are in line with the present study. Sexual dysfunction is considered as one of the main problems of male and female patients who suffer from diabetes. ^31^ However, the present study did not investigate gender differences. 

The present study showed that the mean score of diabetic women in all dimensions of marital adjustment was lower than that of the non-diabetic women. These results are consistent with that of Keyhani et al. who showed a difference in the marital adjustment scores between diabetic and non-diabetic women, so that marital adjustment in non-diabetic women was higher than that in diabetic women. ^32^ To explain the difference in marital adjustment between diabetic women and healthy women, it can be said that chronic diseases like diabetes are an unfortunate event in life that can change the family reactions and interactions. The double burden of responsibility for the spouse due to the disease has an important effect on the quality of life and marital adjustment and relationship. ^33^ Considering the role psychological distress plays in marital adjustment for both women and their partners, couples should be screened for psychological distress after diagnosis and monitored during treatments because Classen et al. suggested that psychosocial support after the diagnosis of chronic disease prevented marital incompatibility. ^34^

The present study indicated that the mean total score of life satisfaction in diabetic women was lower than that of non-diabetic women. Moreover, the total score of life satisfaction in diabetic women was moderate. A study showed moderate life satisfaction in diabetic patients. ^35^ A similar study showed that patients with diabetes and hypertension had lower life satisfaction with long-term disabilities. ^36^ Additionally, a researcher indicated that diabetic women had a lower level of life satisfaction than men. ^15^ However, our results did not reveal gender differences. Shirom showed that poor life satisfaction predicted the incidence of diabetes, ^37^ which may be the reason for poor life satisfaction in diabetic patients. The results of a study showed that psychological and family support was the main key to increased life satisfaction in patients with chronic disease. ^38^ Thus, chronic patients, such as diabetics, should be supported by their families and health workers.

The present study indicated a significant relationship among sexual function, life satisfaction, and marital adjustment. Another study has shown a positive correlation between sexual function and satisfaction with life in middle-aged women with diabetes. ^39^ In addition, another study showed a positive and significant relationship between sexual function and marital adjustment, ^40^ and a positive correlation between marital adjustment and life satisfaction. ^41^ Although the results of the above studies were consistent with those of the current study, healthy women were studied. 

One of the study limitations was lack of cooperation of some patients due to improper physical status and fatigue while filling in the forms. A friendly relationship between the researcher and the patient helped minimize this limitation. This study also had strengths such as the large sample size. In addition, it was a comparative one. 

## CONCLUSION

The results of the study showed that diabetic women experienced sexual dysfunction because of their special condition, which had negative effects on all of their life dimensions, including marital adjustment and life satisfaction. Diabetes prevalence is increasing in Iran and the world, showing its negative effects on the life.

The authors suggest that studies should be conducted on the reason why health care providers have paid less attention to the sexual function of diabetic women. Nurses should prepare educational packages and implement behavioral cognitive therapy intervention to alleviate the problems of this group of patients.
